# Simultaneous Component Analysis of *Akebia quinata* Seeds (Lardizabalaceae) by Ultra-Performance Liquid Chromatography–Tandem Mass Spectrometry for Quality and Cytotoxicity Assessment

**DOI:** 10.3390/plants14050669

**Published:** 2025-02-21

**Authors:** Chang-Seob Seo, Jaemoo Chun, Kwang Hoon Song

**Affiliations:** 1 KM Science Research Division, Korea Institute of Oriental Medicine, Yuseong-daero 1672, Yuseong-gu, Daejeon 34054, Republic of Korea; 2 KM Convergence Research Division, Korea Institute of Oriental Medicine, Yuseong-daero 1672, Yuseong-gu, Daejeon 34054, Republic of Korea; jchun@kiom.re.kr (J.C.); ksong@kiom.re.kr (K.H.S.)

**Keywords:** simultaneous determination, *Akebia quinate* seeds, quality assessment, UPLC–MS/MS, cytotoxicity

## Abstract

*Akebia quinata* seeds (AQSs) are used as an analgesic, antiphlogistic, and diuretic in traditional herbal medicine. We developed an ultra-performance liquid chromatography with tandem mass spectrometry (UPLC–MS/MS) simultaneous component analysis method to analyze eight compounds (chlorogenic acid, isochlorogenic acid A, isochlorogenic acid C, hederacolchiside F, hederacoside C, dipsacoside B, akebia saponin D, and α-hederin) as markers for the quality assessment of AQSs. The separation of the eight analytes was performed in an Acquity UPLC BEH C_18_ reversed-phase analytical column. The method was validated with respect to linearity (coefficient of determination ≥ 0.994), recovery (90.32–108.18%; relative standard deviation (RSD) < 10.0%), and precision (RSD < 10%). The analysis of the AQSs confirmed that the eight components were found in concentrations of 0.42–9.07 mg/g. The cytotoxicity of the AQS extract and the eight compounds against human cancer cell lines, including MDA-MB-231 (breast), A549 (lung), HCT 116 (colon), AsPC-1 (pancreas), and A2780 (ovarian), was also assessed, with cisplatin used as a positive control. In addition, dipsacoside B showed high cytotoxicity in all cell lines. This assay will help to enhance efficacy and clinical research as well as provide a validated quality assessment of AQS extract and related traditional herbal medicines.

## 1. Introduction

*Akebia quinata* (Thunb. Ex Houtt.) Decne. (family: Lardizabalaceae), also known as chocolate vine or five-leaf akebia, is a traditional herbal medicine that grows in East Asia, especially in Korea, Japan, and China [1]. Traditional herbal medicine has been used to prevent and treat diseases through the use of plants or their extracts [1,2]. In ancient societies, it was often closely linked to religious rituals and beliefs [2]. Over time, through empirical observation and scientific research, it played a role in laying the foundation for modern medicine [2]. Today, it continues to be practiced in various forms, such as traditional Korean medicine, traditional Chinese medicine, and Kampo medicine. *A. quinate* is used as a diuretic and tranquilizer to treat hypothermia and rheumatic pain, while the fruit of *A. quinata*, including the seeds, is used as an antitumor agent, analgesic, antiphlogistic, and diuretic [3,4]. Interestingly, its fruits and leaves are registered as raw food materials in a database hosted by the Korean Ministry of Food and Drug Safety, suggesting that they can be used as raw materials for functional food development [4,5,6].

Studies on the biological activity of *A. quinate* have demonstrated the diuretic and neuroprotective effects of the stem extract [7,8], the antiobesity effects of the leaf extract [9], and the hepatoprotective, anti-skin-aging, antiobesity, and hypolipidemic effects of the fruit extract [5,10,11,12]. Recently, the anti-inflammatory and antidiabetic effects of compounds (mainly akebia saponin D and stigmasterol-3-*O*-*β*-_D_-glucoside) isolated from the stem and fruit of *A. quinate* have also been reported [13,14]. In addition, Shida et al. [15] evaluated the inhibitory activity of saponins from *A. quinate* seeds (AQSs) against cells infected with human T-cell leukemia virus type 1.

Various phytochemical components, such as polyphenols [12], steroids [14], triterpenoids [14,16], triterpenoid saponins [15,17,18], coumarins [18], pentacyclic alkaloids [19], essential oils [20], and polysaccharides [21], have been isolated and reported from various parts of *A. quinate*, including the stems, leaves, fruits, and seeds.

Given that *A. quinate* contains numerous characteristic compounds that exhibit various effects, systematic quality control is very important to maintain consistent efficacy and manage the raw materials efficiently. In general, the quality assessment of traditional herbal medicines is performed using high-performance liquid chromatography (HPLC), liquid chromatography with mass spectrometry (LC–MS/MS), gas chromatography, and gas chromatography with mass spectrometry. In the case of *A. quinate*, quality control and phytochemical profiling studies have been conducted using high-performance thin-layer chromatography (HPTLC), HPLC coupled with quadrupole–time-of-flight mass spectrometry (HPLC–Q-TOF–MS/MS), and LC–MS/MS [3,4,22]. Nikolaichuk et al. [3] and Ling et al. [22] performed component profile analyses of the leaves and fruits of *A. quinate* using HPTLC, HPLC-heart-cut-HPLC–MS, and HPLC–Q-TOF–MS/MS. In addition, Gao et al. [4] quantitatively analyzed phenylpropanoids (neochlorogenic acid, chlorogenic acid (CA), cryptochlorogenic acid, and isochlorogenic acid C (ICAC)) and flavonoids (rutin) in leaves using HPLC and LC–MS/MS. However, with the exception of the study by Gao et al. [4], most research has focused on ingredient profiles, and the development of analytical methods for efficient quality control has been limited.

In this study, we focused on developing and validating an ultra-performance liquid chromatography with mass spectrometry (UPLC–MS/MS) multiple reaction monitoring (MRM) simultaneous component analysis method for robust quality control of AQS extract based on eight target components (CA, isochlorogenic acid A (ICAA), ICAC, hederacolchiside F (HF), hederacoside C (HC), dipsacoside B (DB), akebia saponin D (ASD), and α-hederin (HDR)). The method was validated with respect to linearity, sensitivity, accuracy, and precision. Furthermore, the cytotoxicity of the AQS extract and the eight analytes against various human cancer cell lines, including MDA-MB-231 (breast), A549 (lung), HCT 116 (colon), AsPC-1 (pancreas), and A2780 (ovarian) cells, was evaluated.

## 2. Results

### 2.1. Analysis Conditions for Quality Assessment of AQS Samples by UPLC–MS/MS

Simultaneous analysis was performed using the eight target components (CA, ICAA, ICAC, HF, HC, DB, ASD, and HDR) to assess the quality of the AQS samples using the UPLC–MS/MS system.

**Figure 1:**
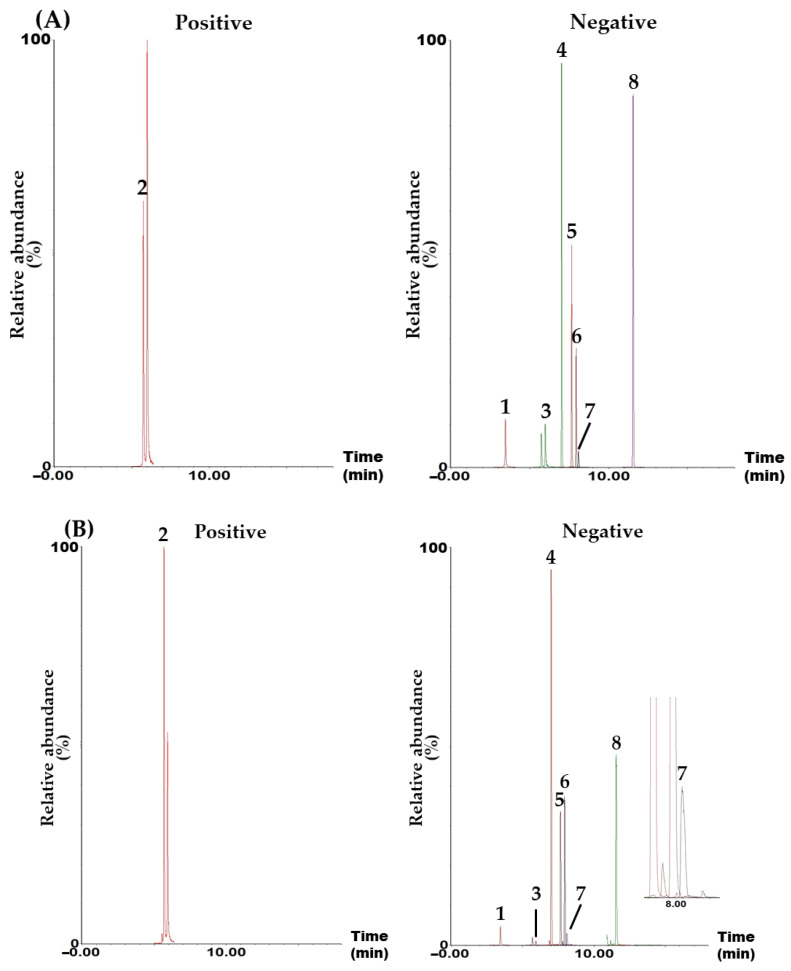
Representative total ion chromatograms of the standard solution containing the eight target analytes (**A**) and 70% ethanol extract of the AQS sample (**B**) measured in positive and negative ion modes using the UPLC–MS/MS MRM analytical method. Chlorogenic acid (CA, 1), isochlorogenic acid A (ICAA, 2), isochlorogenic acid C (ICAC, 3), hederacolchiside F (HF, 4), hederacoside C (HC, 5), dipsacoside B (DB, 6), akebia saponin D (ASD, 7), and α-hederin (HDR, 8). The concentration of each analyte in the mixed standard solution was 2400 μg/L for CA; 1920 μg/L for ICAA; 4000 μg/L for ICAC; 1600 μg/L for HF, HC, and ASD; 960 μg/L for DB; and 640 μg/mL for HDR.

Simultaneous component analysis was performed using a UPLC–MS/MS system (Waters, Milford, MA, USA) consisting of an Acquity UPLC I-Class Plus (Waters) and a Xevo TQ-S micro tandem quadrupole mass spectrometer (Waters). This system included an electrospray ionization ion source and an MRM mode, which is widely used for quantitative analysis in various fields, such as omics, metabolics, and natural products, due to its high selectivity, specificity, and sensitivity. The target compounds were separated in a Waters Acquity UPLC BEH C_18_ reversed-phased analytical column (130 Å, 2.1 mm × 100 mm, 1.7 μm, part no. 186002352) using a gradient elution with a mobile phase consisting of distilled water and acetonitrile (both containing 0.1% formic acid; Table S1). All target analytes were detected in either negative (CA, ICAC, HF, HC, DB, ASD, and HDR) or positive (ICAA) ion modes within 12.0 min (Figure 1 and Figure S1).

### 2.2. Identification of Eight Target Compounds in UPLC–MS/MS MRM Mode for Simultaneous Component Analysis

To simultaneously analyze the eight target compounds selected for the quality control of AQSs, the MRM conditions (precursor ion [Q1], product ion [Q3], cone voltage, and collision energy) for each compound were established, as shown in Table 1. The Q3 peaks of the phenylpropanoids, CA, ICAA, and ICAC, were set to *m*/*z* 190.94, 116.96, and 172.98 in the form of [quinic acid–H]^–^, [caffeoyl group+H–CO–H_2_O]^+^, and [quinic acid–H–H_2_O]^–^, respectively. Briefly, the Q1 peaks of CA and ICAC were observed at *m*/*z* 352.99 and 515.02 in negative-ion mode. The Q3 peak of CA (*m*/*z* 190.94) was derived from quinic acid generated by the cleavage of the ester bond between quinic acid and caffeic acid moieties, and the Q3 of ICAC was generated by the removal of one water molecule from quinic acid [23,24]. The Q3 peak of ICAA detected at *m*/*z* 517.08 (Q1) in the positive-ion mode was generated by the removal of CO and H_2_O molecules from the caffeoyl group [24]. The Q3 peaks of HF and HC were detected at *m*/*z* 911.29 and 749.31, respectively, which were formed by the removal of one molecule of rhamnose and two molecules of glucose from their Q1 peaks to form [M–Rha–2Glu–H]^–^ [25,26]. The Q1 peaks of DB and ASD were detected at *m/z* 1073.52 [M–H]^–^ and 973.41 [M+HCOO]^–^, respectively. Their Q3 peaks were detected at *m/z* 749.31 and 603.23 (both [M–H–2Glu]^–^), where two glucose groups were removed [27,28]. Finally, for HDR, the Q1 peak was detected at *m/z* 749.29 [M–H]^–^, and the Q3 peak was observed at *m/z* 471.22 [M–H–Rha–Arb]^–^ in the aglycone form with the rhamnose–arabinose group removed from the Q1 peak [27].

### 2.3. Method Validation of Developed UPLC–MS/MS MRM Assay

The developed UPLC–MS/MS MRM assay was validated according to the International Conference on Harmonization (ICH) guidelines [29], namely, linearity, the limit of quantitation (LOQ), accuracy (recovery), and precision were evaluated. As shown in Table 2, the *r*^2^ value, which indicates linearity in the regression equation of each compound measured at different concentrations, was ≥0.994, showing that the linearity of the calibration curve measured was appropriate. The LOQ concentration (signal-to-noise ratio [S/N] ≥ 10) was set as the lowest concentration of the calibration curve for each target; the LOQ values for the AQS samples are shown in Table 2. The recovery of the eight target compounds was 90.32–108.18%, showing satisfactory recovery within the acceptable range of ±15% (Table 3). The precision of the method was evaluated using the relative standard deviation (RSD) of the results measured within 1 day and for 3 consecutive days; the RSD value was ≤8.56%, showing an appropriate result (Table 4). The repeatability of the method (RSD < 8.0%) was evaluated by recording the retention time and peak area of each compound six times (Table S2). Based on the above validation data, the UPLC–MS/MS MRM analytical method developed for the quality control of the AQS samples was shown to be appropriate.

### 2.4. Simultaneous Determination of Eight Target Compounds in AQS Sample by UPLC–MS/MS MRM

The validated UPLC–MS/MS method was successfully applied to the simultaneous quantitative analysis of a lyophilized AQS sample. The eight target compounds were detected at concentrations of 0.42–9.07 mg/g, with DB (7.04 mg/g) and ASD (9.07 mg/g) found in relatively high abundances compared with the other compounds. The quantitative results are summarized in Table 5.

### 2.5. Cytotoxic Effects on Cancer Cell Lines of AQS Extract and Eight Compounds

**Figure 2:**
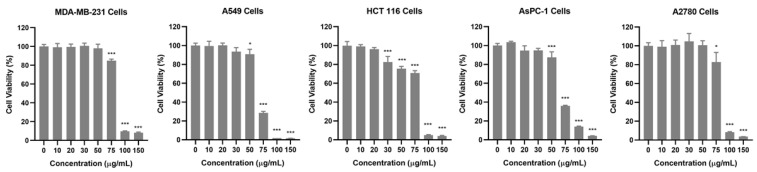
Cytotoxic effects of AQS extract on cancer cell lines. MDA-MB-231, A549, HCT 116, AsPC-1, and A2780 cells were treated with AQS extract (0–150 μg/mL) for 24 h. Cell viability was assessed using a CCK-8 assay. Bars represent the means ± SDs. * *p* < 0.05 and *** *p* < 0.001 indicate a significant difference compared with the control (0 μg/mL) group.

To investigate the anticancer potential of the AQS extract, its cytotoxic effects were evaluated in five human cancer cell lines: breast (MDA-MB-231), lung (A549), colon (HCT 116), pancreas (AsPC-1), and ovarian (A2780). Cell viability was assessed using the Cell Counting Kit-8 (CCK-8, Dojindo, Tokyo, Japan) assay after treating the cells with various concentrations of the AQS extract for 24 h. As shown in Figure 2 and Figure S2, the AQS extract treatment resulted in a dose-dependent reduction in all tested cancer cell lines. Among the tested concentrations, A549 and AsPC-1 cells displayed a significant reduction at 75 µg/mL, whereas MDA-MB-231, HCT 116, and A2780 cells required higher concentrations to achieve similar effects. These results indicate that the AQS extract has broad-spectrum cytotoxic activity against diverse cancer cell types, with notable potency against A549 and AsPC-1 cells.

To identify the specific bioactive components responsible for the cytotoxicity of the AQS extract, eight compounds, CA, ICAA, ICAC, HF, HC, DB, ASD, and HDR, from the AQS extract were tested for their anticancer effects. Each compound was evaluated for its cytotoxicity in the same five cancer cell lines (MDA-MB-231, A549, HCT 116, AsPC-1, and A2780) using the CCK-8 assay. Among these, DB demonstrated the strongest cytotoxic activity, showing significant cytotoxic effects on all five cancer cell lines. Notably, DB exhibited the greatest cytotoxicity in A549 cells, with a half-maximal inhibitory concentration (IC_50_) value of 11.89 ± 0.23 μM, which was the lowest among the tested cell lines. The IC_50_ values for DB in the other cell lines were determined to be 15.15 ± 0.20 μM (MDA-MB-231), 14.27 ± 1.00 μM (HCT 116), 13.12 ± 0.26 μM (AsPC-1), and 17.21 ± 0.35 μM (A2780). In contrast, the other compounds did not show significant cytotoxicity against the cancer cell lines tested (Table 6 and Figure S2). These results indicate that DB is the primary bioactive compound in the AQS sample, contributing significantly to its anticancer properties.

## 3. Discussion

In this study, a UPLC–MS/MS MRM assay for the simultaneous determination of the main constituents in an AQS sample was developed. A variety of components, including polyphenols (e.g., CA, ICAA, and ICAC) [11], steroids (e.g., stigmasterol-3-*O*-β-_D_-glucoside) [13], triterpenoids (e.g., maslinic acid (MA), scutellaric acid (SA), and hederagenin (HDRG)) [13], triterpenoid saponins (e.g., akebia saponin PA (ASPA), HDR, HC, and HF) [2,13], coumarins (e.g., aesculetin and hymexelsin) [17], pentacyclic alkaloids (e.g., ormosanine) [18], and essential oils (e.g., limonene and palmitic acid) [19] were isolated and analyzed from various parts of *A. quinate*. Among the numerous reported ingredients, we attempted the simultaneous determination of the first 12 components using the UPLC–MS/MS MRM assay. The 12 tested components were CA, ICAA, ICAC, HF, HC, DB, ASD, HDR, SA, MA, HDRG, and ASPA. However, four of the compounds (SA, MA, HDRG, and ASPA) were not detected in the AQS sample. Therefore, these four compounds were excluded, and the remaining compounds were used as target compounds for the quality assessment of the AQS sample (Figure 1).

A simultaneous analysis method for the eight marker compounds was successfully developed using gradient elution with a mobile phase consisting of water and acetonitrile, both containing 0.1% formic acid. Furthermore, the developed analytical method was validated for its suitability by evaluating linearity, sensitivity (LOQ), recovery, and precision (Table 2, Table 3 and Table 4). Using this assay, all markers were detected within 12.0 min (Figure 1), with ASD being the most abundant in the AQS sample, at 9.07 ± 0.33 mg/g (Table 5).

Our study confirmed that DB exhibits potent cytotoxicity against multiple cancer cell lines, suggesting that it may be a key bioactive component contributing to the anticancer properties of AQSs. DB is an oleanane-type triterpenoid saponin with various pharmacological activities. Recent studies have shown that DB attenuates atherosclerosis [30], alleviates cardiovascular disease [31], improves cognitive dysfunction [32], and provides neuroprotection against ischemic stroke [33]. Additionally, oleanane-type saponins exert anticancer effects through multiple pathways, including the induction of apoptosis, inhibition of metastasis, and modulation of STAT3/MAPK signaling [34]. Based on these findings and our results, we propose that DB has significant potential for application in cancer therapy, further supporting its role as a key bioactive compound in AQSs.

Several compounds analyzed in this study have been previously reported for their anticancer activity. CA has been shown to possess anticancer properties, including the inhibition of cancer growth through multiple pathways, such as cell cycle arrest and oxidative stress modulation [35,36]. Moreover, saponins, including HDR, are known for their potential anticancer effects, as they can enhance apoptosis [37]. These compounds regulate key cancer-related pathways such as PI3K/Akt and NF-κB, leading to apoptosis and the inhibition of cancer cell proliferation [38]. However, CA derivatives exhibited only slight inhibition of cell proliferation under the experimental conditions and concentrations used. Additionally, other saponins, except DB, did not show cytotoxicity despite their structural similarity. These findings highlight the need for further investigation into the structure–activity relationship and molecular mechanisms underlying DB, including its interactions with cancer-specific signaling pathways and its role in inducing cell death mechanisms. A deeper understanding of these mechanisms could reveal potential therapeutic targets and further enhance the efficacy of AQS-based treatments.

One major strength of this study was the use of a validated UPLC-MS/MS analytical method, which ensured the accurate quantification of the active compounds in the AQSs. Additionally, the cytotoxicity of DB was assessed using multiple cancer cell lines, increasing the reliability of our findings. However, skepticism remains among clinicians regarding the integration of natural compounds into standard cancer therapies. Many oncologists are concerned about the lack of clinical trial data and the standardization issues associated with herbal-based therapies [39]. Our results provide valuable insights into the standardization of AQS extract, demonstrating the reproducibility and consistency of DB’s cytotoxic effects. Nevertheless, further studies are necessary to explore the pharmacokinetics, bioavailability, and toxicity profiles of AQS extract and DB in vivo. Assessing their efficacy and safety in animal models will be essential before considering clinical trials. Additionally, the interaction between DB and other compounds present in AQS extract should be investigated, as their potential synergistic effects may enhance cytotoxicity and provide additional therapeutic benefits.

## 4. Materials and Methods

### 4.1. Plant Materials

AQS samples were provided by Dr. Changjin Kim, Woorimadi Lapa Medi Lab (Jeonju, Republic of Korea). These raw materials were used for research after morphological identification by Dr. Goya Choi, a herbalist at the Korea Institute of Oriental Medicine (Naju, Republic of Korea). A voucher specimen (AQSE-2021) was stored at the KM Science Research Division, the Korea Institute of Oriental Medicine (KIOM, Daejeon, Republic of Korea).

### 4.2. Chemicals and Reagents

The eight reference standards used in this study were purchased from manufacturers specializing in natural products: chlorogenic acid was obtained from Merck KGaA (Darmstadt, Germany); ICAA and ICAC from Shanghai Sunny Biotech (Shanghai, China); HF, HC, and HDR from Biopurify Phytochemicals (Chengdu, China); DB from ChemFaces Biochemical (Wuhan, China); and ASD from BioFron (Fullerton, CA, USA) (Table S3 and Figure S3). Methanol, acetonitrile, and formic acid were all LC–MS-grade and purchased from JT Baker (Phillipsburg, NJ, USA) and Thermo Fisher Scientific (Cleveland, OH, USA). Ultrapure deionized water was generated using a Milli-Q Integral 15 (Millipore, Molsheim, France) and an Elix Technology Inside system with a resistivity of at least 15.0 MΩ·cm. Roswell Park Memorial Institute (RPMI) 1640 medium and fetal bovine serum (FBS) were purchased from Corning (Corning, NY, USA). Penicillin and streptomycin were purchased from Gibco (Grand Island, NY, USA). Cisplatin was provided by Sigma-Aldrich (St. Louis, MO, USA).

### 4.3. Preparation of 70% Ethanol Extract of AQSs

The 70% ethanolic AQS extract was manufactured by KIOM. In detail, 500 mL of 70% ethanol was added to 300 g of dried and ground AQSs, and ultrasonic extraction was performed for 1 h at room temperature. After filtering the extract solution, the above process was repeated three times. The filtered extract solution was centrifuged at 3000 rpm for 15 min, and the organic solvent was removed from the supernatant using a rotary evaporator system (R-210, Büchi, Flawil, Switzerland). The concentrated residue was suspended in 500 mL of distilled water, frozen, and then lyophilized using a freeze-dryer (FD8518, IlShinBioBase, Yangju, Republic of Korea) to obtain a powder sample (21.6 g; 7.2% yield). The prepared sample was stored at −20 °C and used for analysis.

### 4.4. Preparation of Standard and Sample Solutions for UPLC–MS/MS Analysis

A standard solution of each reference standard compound was prepared at a concentration of 1.0 mg/mL using methanol. The standard solutions were stored frozen (−20 °C) and used for analysis when necessary. The sample solution for UPLC–MS/MS analysis was prepared by adding 10 mL of 50% methanol to 100 mg of the freeze-dried AQS sample, followed by ultrasonic extraction for 10 min and subsequent vortexing for 10 min. For quantification, the prepared sample solution was diluted as follows: CA and ICAA were diluted 10-fold, and HF, HC, DB, ASD, and HER were diluted 100-fold before measurement. All solutions were filtered through a 0.2 μm membrane filter (GVS ABLUO, Sandford, ME, USA) and then used for analysis.

### 4.5. Analytical Conditions for Simultaneous Determination of Eight Target Analytes in AQS Sample by UPLC–MS/MS

The simultaneous determination of the eight analytes in the AQS sample was performed using a UPLC–MS/MS system (Waters) combining an Acquity UPLC I-Class Plus (Waters) and a Xevo TQ-S micro triple quadrupole mass system (Waters). The operating conditions for the simultaneous determination are shown in Table S1. In particular, the MRM mode was used, which has the advantages of excellent sensitivity, selectivity, and high precision for the quantification of target compounds in complex samples. Various parameters (e.g., MRM transition, cone voltage, and collision energy) for the MRM analysis are shown in Table 1.

### 4.6. Validation of the UPLC–MS/MS MRM Assay

The developed UPLC–MS/MS MRM assay was validated for linearity, LOQ, accuracy (recovery), and precision based on the ICH guidelines [29]. In other words, linearity was evaluated by the *r*^2^ value in the calibration curve of each target compound, and the lowest concentration in the range of the calibration curve was set as the LOQ concentration. The LOQ concentration was validated by confirming that the S/N of each target compound was ≥10 at the set LOQ concentration.

The accuracy (recovery %) was validated by the standard addition method. This method involves adding three different concentrations (low, medium, and high) to a known actual sample and measuring their concentrations. The recovery was calculated using the following equation:

$$Recovery\left(\%\right)=\frac{found\;amount}{spiked\;amount}\times 100$$

The precision was evaluated by RSD (%) values after measuring three different concentrations for 1 day and 3 consecutive days. The reproducibility was also evaluated by the RSD (%) values after measuring six times repeatedly on the same day using solutions containing the eight target compounds. The RSD (%) was calculated using the following equation:

$$RSD\left(\%\right)=\frac{standard\;deviation}{mean}\times 100$$

### 4.7. Cytotoxicity Tests

#### 4.7.1. Cell Culture

MDA-MB-231, A549, HCT 116, and AsPC-1 cells were obtained from the Korean Cell Bank (Seoul, Republic of Korea). A2780 cells were obtained from the European Collection of Authenticated Cell Cultures (Salisbury, UK). The cells were grown in RPMI 1640 medium supplemented with 10% FBS, 100 U/mL penicillin, and 100 μg/mL streptomycin. The cells were maintained at 37 °C in a humidified atmosphere with 5% CO_2_.

#### 4.7.2. Cell Proliferation Assay

The cell proliferation was determined using CCK-8 according to the manufacturer’s instructions. Briefly, MDA-MB-231, A549, HCT 116, AsPC-1, and A2780 cells were seeded into a 96-well plate at a density of 1 × 10^4^ cells/well. The following day, the cells were treated with various concentrations of the extracts and compounds. After 24 h of treatment, 10 μL of CCK-8 reagent was added to each well, and the plates were incubated for an additional 3 h at 37 °C. The absorbance of the CCK-8 reagent was measured at 450 nm using a SpectraMax i3 microplate reader (Molecular Devices, San Jose, CA, USA). Cisplatin was used as a positive control. Dose–response curves were generated to calculate the IC_50_ values using GraphPad Prism 9 (San Diego, CA, USA).

### 4.8. Statistical Analysis

All data are presented as means ± SDs from three independent experiments. The statistical significance between groups was evaluated using a one-way analysis of variance followed by Dunnett’s post hoc test using GraphPad Prism 9. A *p*-value of <0.05 was considered statistically significant.

## 5. Conclusions

In this study, the UPLC–MS/MS MRM analytical method was developed and validated for the efficient quality control of AQS samples. The analytical method was validated by examining its linearity, LOQ, accuracy (recovery), and precision. Among the eight compounds selected for quality control, DB and ASD were found to be the most abundant. In particular, DB showed strong cytotoxicity in various anticancer cell lines, including MDA-MB-231, A549, HCT116, AsPC-1, and A2780, emphasizing the importance of further studies to elucidate its molecular mechanism. These findings can contribute to evaluating the efficacy and safety of AQS extract for potential clinical applications.

## Figures and Tables

**Table 1:** The parameters for the simultaneous quantification of the eight target compounds using the UPLC–MS/MS MRM method.

Analyte ^1^	Ion Mode	Molecular Weight	MRM Conditions (*m/z*)		Cone Voltage (V)	Collision Energy (eV)
			Precursor Ion (Q1)	Product Ion (Q3)		
CA	−	354.10	352.99	190.94	22	14
ICAA	+	516.13	517.08	116.96	10	58
ICAC	−	516.13	515.02	172.98	12	30
HF	−	1382.67	1381.51	911.29	96	44
HC	−	1220.62	1219.66	749.31	76	68
DB	−	1074.56	1073.52	749.31	76	62
ASD	−	928.50	973.41	603.23	2	54
DER	−	750.46	749.29	471.22	98	46

^1^ Chlorogenic acid (CA), isochlorogenic acid A (ICAA), isochlorogenic acid C (ICAC), hederacolchiside F (HF), hederacoside C (HC), dipsacoside B (DB), akebia saponin D (ASD), and α-hederin (HDR).

**Table 2:** Retention time, linear range, regression equation, coefficient of determination (*r*^2^), and limit of quantitation (LOQ) values of the target compounds measured using the UPLC–MS/MS MRM method.

Analyte	Retention Time (min)	Linear Range (μg/L)	Regression Equation ^1^ y=ax+b	*r* ^2^	LOQ (mg/g)
CA	3.44	150.00–2400.00	*y* = (1.45 ± 0.12)*x* + (4.68 + 2.81)	0.999	0.15
ICAA	5.73	120.00–1920.00	*y* = (8.08 ± 0.59)*x* + (114.96 ± 23.02)	0.999	0.12
ICAC	5.97	250.00–4000.00	*y* = (0.37 ± 0.01)*x* − (24.95 ± 3.92)	0.994	0.03
HF	7.00	100.00–1600.00	*y* = (12.05 ± 0.58)*x* − (165.78 ± 30.54)	0.999	1.00
HC	7.65	100.00–1600.00	*y* = (4.05 ± 0.10)*x* − (58.60 ± 8.21)	1.000	1.00
DB	7.95	120.00–1920.00	*y* = (5.63 ± 0.15)*x* − (28.44 ± 7.91)	0.999	1.20
ASD	8.09	100.00–1600.00	*y* = (0.47 ± 0.02)*x* + (19.37 ± 0.84)	0.996	1.00
DER	11.54	40.00–640.00	*y* = (23.44 ± 0.45)*x* + (153.22 ± 25.66)	0.999	0.40

^1^*y*: peak area of compounds; *x*: concentration (μg/L) of compounds. Data are presented as means ± standard deviations (SDs).

**Table 3:** Recovery results of the eight analytes for the developed UPLC–MS/MS MRM method (n = 3).

Analyte	Spiked Amount (μg/L)	Found Amount (μg/L)	Recovery (%)	SD ^1^	RSD (%) ^2^
CA	400.00	409.02	102.26	1.57	0.38
	500.00	497.70	99.54	12.47	2.50
	600.00	571.37	95.23	5.66	0.99
ICAA	720.00	705.81	98.03	26.30	3.73
	900.00	973.59	108.18	66.73	6.85
	1200.00	1264.99	105.42	91.94	7.27
ICAC	600.00	575.21	95.87	42.79	7.44
	1200.00	1263.51	105.29	78.37	6.20
	1800.00	1864.78	103.60	28.42	1.52
HF	620.00	588.85	94.98	11.37	1.93
	820.00	828.88	101.08	4.36	0.53
	1020.00	1023.89	100.38	17.51	1.71
HC	600.00	575.21	95.87	42.79	7.44
	1200.00	1263.51	105.29	78.37	6.20
	1800.00	1864.78	103.60	28.42	1.52
DB	1000.00	903.17	90.32	35.87	3.97
	1300.00	1225.91	94.30	41.61	3.39
	1600.00	1496.78	93.55	11.90	0.80
ASD	850.00	776.04	91.30	32.82	4.23
	1000.00	921.98	92.20	30.18	3.27
	1150.00	1068.74	92.93	39.77	3.72
HDR	400.00	409.47	102.37	5.75	1.40
	550.00	581.04	105.64	6.29	1.08
	700.00	721.01	103.00	10.04	1.39

^1^ SD: standard deviation. ^2^ RSD: relative standard deviation.

**Table 4:** Precision of the developed UPLC–MS/MS MRM method for the eight target compounds.

Analyte	Conc. (μg/L)	Intraday (n = 3)				Interday (n = 15)	
		Observed Conc. (μg/L)	Precision (RSD, %)	Accuracy (%)	Observed Conc. (μg/L)	Precision (RSD, %)	Accuracy (%)
CA	300.00	295.04	0.18	98.35	300.03	4.93	100.01
	600.00	626.49	6.90	104.42	603.11	2.32	100.52
	1200.00	1230.15	3.33	102.51	1177.18	2.43	98.10
ICAA	120.00	112.96	6.04	94.13	111.92	3.52	93.27
	240.00	241.57	2.69	100.65	246.68	0.64	102.78
	480.00	483.75	2.00	100.78	503.94	4.04	104.99
ICAC	250.00	253.33	4.97	101.33	269.23	7.19	107.69
	500.00	504.64	0.80	100.93	481.62	0.97	96.32
	100.00	1004.15	1.92	100.41	997.15	8.56	99.71
HF	400.00	437.71	4.31	109.43	387.11	1.95	96.78
	800.00	806.07	4.75	100.76	792.91	0.80	99.11
	1600.00	1492.89	2.02	93.31	1618.89	0.07	101.18
HC	100.00	109.18	5.47	109.18	102.89	2.63	102.89
	200.00	198.16	4.43	99.08	199.40	1.07	99.70
	400.00	378.70	2.87	94.68	387.20	3.25	96.80
DB	240.00	234.39	6.20	97.66	243.50	4.18	101.46
	480.00	468.92	2.22	97.69	481.13	4.74	100.23
	960.00	1003.14	5.04	104.49	943.88	2.18	98.32
ASD	400.00	379.59	4.33	94.90	421.64	3.05	105.41
	800.00	824.79	4.72	103.10	846.66	6.96	105.83
	1600.00	1589.68	1.85	99.36	1540.12	4.04	96.26
HDR	80.00	75.62	5.14	94.53	81.27	1.71	101.58
	160.00	157.46	3.36	98.41	167.06	1.32	104.41
	320.00	330.42	1.70	103.26	329.38	0.30	102.93

**Table 5:** The amounts (mg/g) of the eight target analytes in a lyophilized AQS sample by the UPLC–MS/MS MRM method (n = 3).

Analyte	Mean (mg/g)	SD	RSD (%)
CA	1.35	0.12	9.17
ICAA	0.69	0.07	9.48
ICAC	0.42	0.04	9.01
HF	4.28	0.10	2.29
HC	2.78	0.16	5.67
DB	7.04	0.24	3.43
ASD	9.07	0.33	3.69
DER	2.54	0.03	1.02

**Table 6:** IC_50_ values of compounds in various human cell lines (n = 3).

Compound	IC_50_ Value (μM) ^2^				
	MDA-MB-231	A549	HCT 116	AsPC-1	A2780
DB	15.15 ± 0.20	11.89 ± 0.23	14.27 ± 1.00	13.12 ± 0.26	17.21 ± 0.35
Cisplatin ^1^	39.65 ± 12.44	21.12 ± 8.93	19.72 ± 1.28	19.38 ± 4.03	24.32 ± 2.16

^1^ Cisplatin: positive control. ^2^ Data are presented as means ± SDs.

## Data Availability

The data presented in this study are available in this article (see tables and figures).

## Supplementary Materials

The following supporting information can be downloaded at https://www.mdpi.com/article/10.3390/plants14050669/s1. Figure S1: Extracted ion chromatograms of each standard compound (A) and a 70% ethanol extract of AQS sample (B) by the UPLC–MS/MS MRM method in positive- or negative-ion modes; Figure S2: IC_50_ values of AQS extract (A), each marker compound (B–I), and positive control (J) in various human cell lines. AQS extract (A), chlorogenic acid (B), isochlorogenic acid A (C), isochlorogenic acid C (D), hederacolchiside F (E), hederacoside C (F), dipsacoside B (G), akebia saponin D (H), α-hederin (I), and positive control (cisplatin, J); Figure S3: Chemical structures of the eight target compounds selected for the quality assessment of the AQS sample; Table S1: Operating conditions for the UPLC–MS/MS MRM analysis of the 70% ethanol extract of AQSs; Table S2: Reproducibility for retention times and peak areas of each compound (n = 6); Table S3: Information about the eight reference standard compounds.
