# Supplementary material for: Simultaneous Component Analysis of Akebia quinata Seeds (Lardizabalaceae) by Ultra-Performance Liquid Chromatography–Tandem Mass Spectrometry for Quality and Cytotoxicity Assessment
Source: Plants (Basel). 2025 Feb 21;14(5):669. doi: 10.3390/plants14050669 (PMC11901900; doi:10.3390/plants14050669)
Supplement: Supplementary file 1 [file plants-14-00669-s001.zip › plants-3465886-supplementary.pdf]

Table S1

Operating conditions for the UPLC–MS/MS MRM analysis of 70% ethanol extract of AQS.

UPLC conditions		MS conditions	
UPLC system	Acquity UPLC I-Class Plus	MS system	Xevo TQ-S micro
Column	Acquity UPLC BEH C_{18} column (2.1 mm × 100 mm, 1.7 μm)	MS software	MassLynx v4.2
Column temp.	40 °C	Ion source	ESI^+ or ESI^-
Sample temp.	5 °C	Acquisition mode	MRM
Injection volume	2.0 μL	Capillary voltage	3.3 kV
Flow rate	0.3 mL/min	Cone gas flow	80 L/h
Mobile phase A	0.1% (v/v) aqueous formic acid	Desolvation gas flow	600 L/h
Mobile phase B	0.1% (v/v) formic acid in acetonitrile	Desolvation temp.	300 °C
Gradient program	(see below)	Source temp.	150 °C

Gradient program:

Time (min)	A (%)	B (%)
Initial	95	5
11.4	40	60
14.3	5	95
15.7	5	95
17.1	95	5
20.0	95	5

ESI; electrospray ionization, MRM; multiple reaction monitoring

Table S2

Reproducibility for retention times and peak areas of each compound (n = 6).

Compound[1]	Retention time			Peak area		
	Average	SD ($\times 10^{-1}$)	RSD (%)	Average	SD	RSD (%)
CA	3.44	0.04	0.11	203.55	9.19	4.52
ICAA	5.73	0.03	0.05	1049.78	67.95	6.47
ICAC	5.98	0.03	0.05	71.16	5.66	7.96
HF	7.00	0.04	0.05	1126.85	32.49	2.88
HC	7.65	0.04	0.05	389.34	14.78	3.80
DB	7.93	0.09	0.11	666.50	37.98	5.70
ASD	8.09	0.06	0.08	66.13	3.23	4.88
DER	11.54	0.04	0.03	1021.71	31.26	3.06

[1] Chlorogenic acid (CA), isochlorogenic acid A (ICAA), isochlorogenic acid C (ICAC), hederacolchiside F (HF), hederacoside C (HC), dipsacoside B (DB), akebia saponin D (ASD), and α-hederin (HDR).

Table S3

Information about the eight reference standard compounds.

Analyte	Purity (%)	Molecular formula	CAS No.	PubChem CID	Catalog No.	Maker
CA	99.7	$C_{16}H_{18}O_9$	327-97-9	1794427	PHL89175	Merck KGaA
ICAA	98.2	$C_{25}H_{24}O_{12}$	2450-53-5	6474310	DR11570	Shanghai Sunny Biotech
ICAC	99.9	$C_{25}H_{24}O_{12}$	32451-88-0	6473409	DR11571	Shanghai Sunny Biotech
HF	99.9	$C_{65}H_{106}O_{31}$	68027-14-5	102594492	BP1179	Biopurify Phytochemicals
HC	99.5	$C_{59}H_{96}O_{26}$	14216-03-6	11491905	BP0708	Biopurify Phytochemicals
DB	98.1	$C_{53}H_{86}O_{22}$	33289-85-9	21627940	CFN99150	Wuhan ChemFaces Biochemical
ASD	98.9	$C_{47}H_{76}O_{18}$	39524-08-8	14284436	BF-A1010	BioFron
DER	99.6	$C_{41}H_{66}O_{12}$	27013-91-8	73296	BP0154	Biopurify Phytochemicals

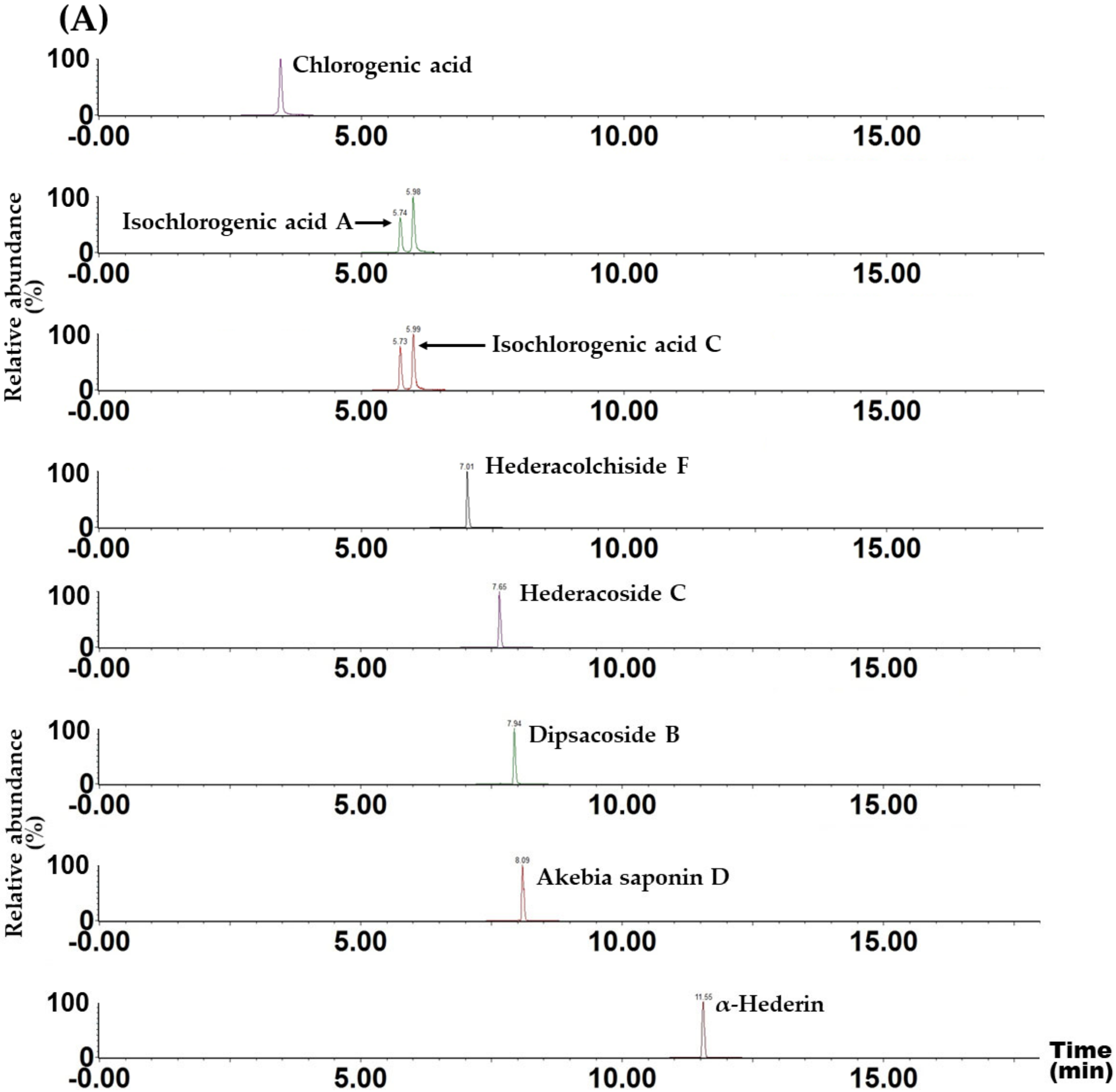
(A)
Chlorogenic acid
Isochlorogenic acid A
Isochlorogenic acid C
Hederacolchiside F
Hederacoside C
Dipsacoside B
Akebia saponin D
α-Hederin
Relative abundance (%)
Time (min)
5.74
5.98
5.73
5.99
7.01
7.65
7.94
8.09
11.55
100
0
-0.00
5.00
10.00
15.00

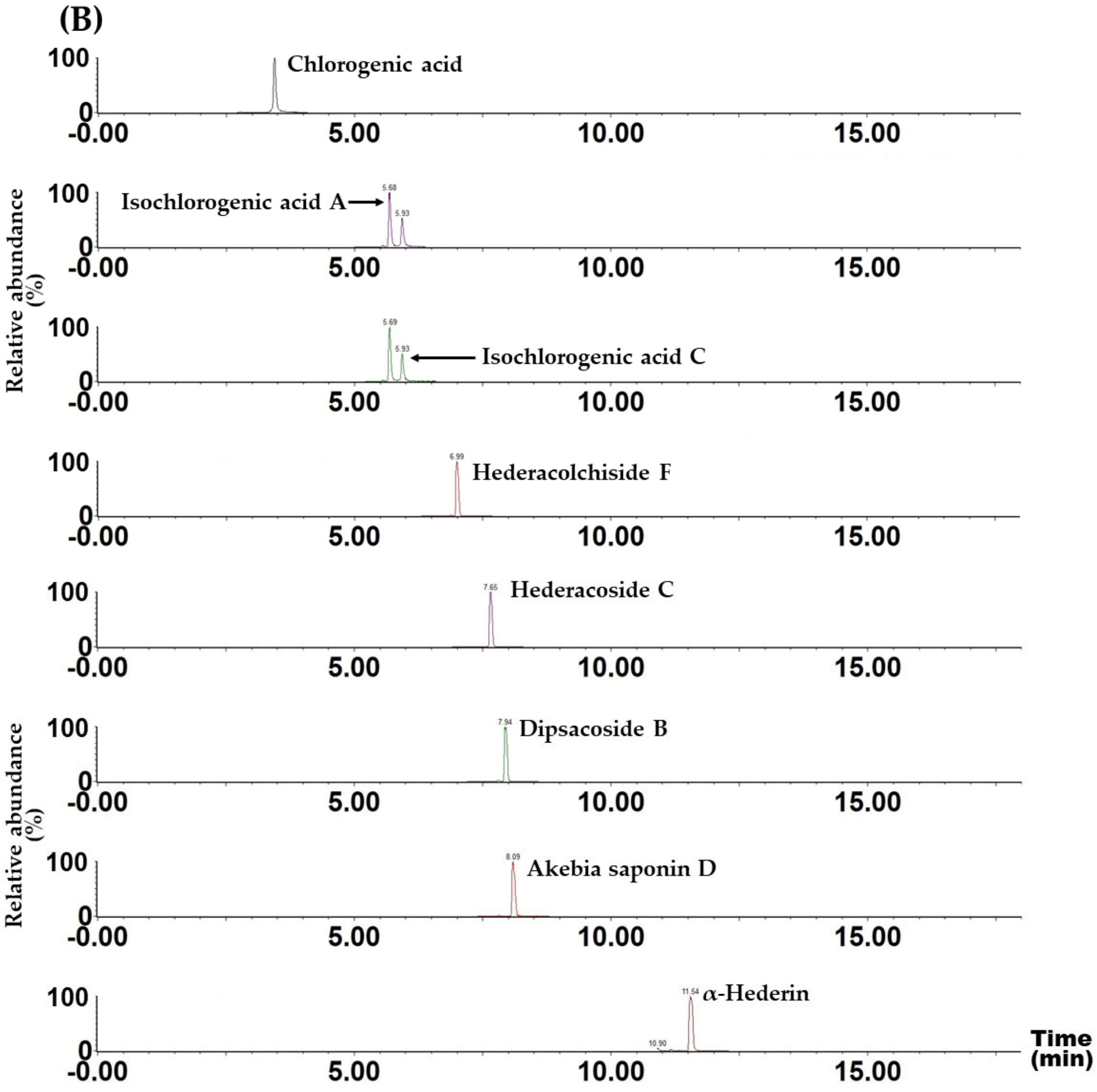

Figure S1. Extracted ion chromatograms of each standard compound (A) and of a 70% ethanol extract of AQS sample (B) by the UPLC–MS/MS MRM method in positive or negative ion modes.

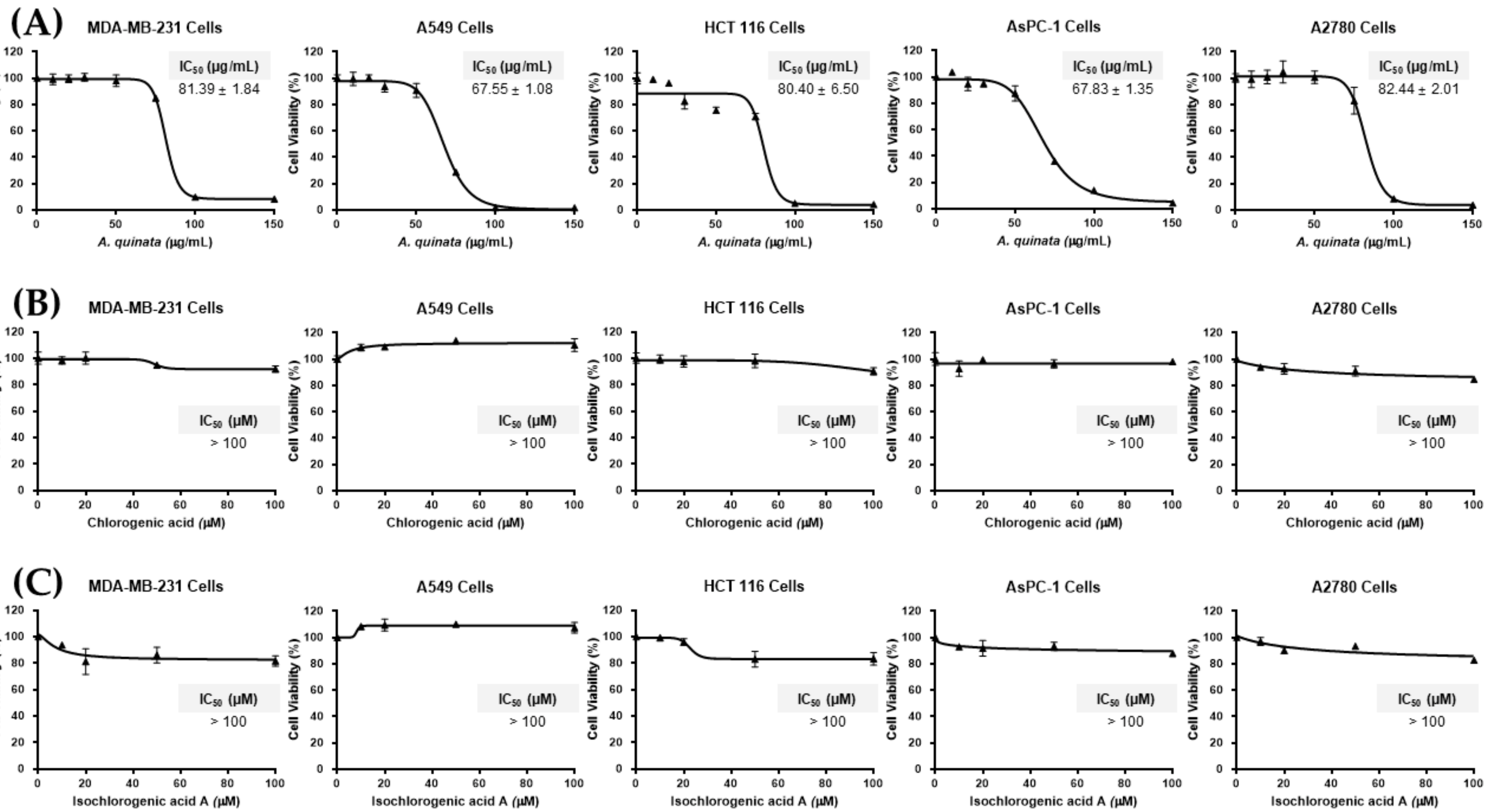
(A)
MDA-MB-231 Cells
A549 Cells
HCT 116 Cells
AsPC-1 Cells
A2780 Cells
IC50 (μg/mL)
81.39 ± 1.84
67.55 ± 1.08
80.40 ± 6.50
67.83 ± 1.35
82.44 ± 2.01
Cell Viability (%)
A. quinata (μg/mL)
(B)
MDA-MB-231 Cells
A549 Cells
HCT 116 Cells
AsPC-1 Cells
A2780 Cells
IC50 (μM)
> 100
Cell Viability (%)
Chlorogenic acid (μM)
(C)
MDA-MB-231 Cells
A549 Cells
HCT 116 Cells
AsPC-1 Cells
A2780 Cells
IC50 (μM)
> 100
Cell Viability (%)
Isochlorogenic acid A (μM)

(D)

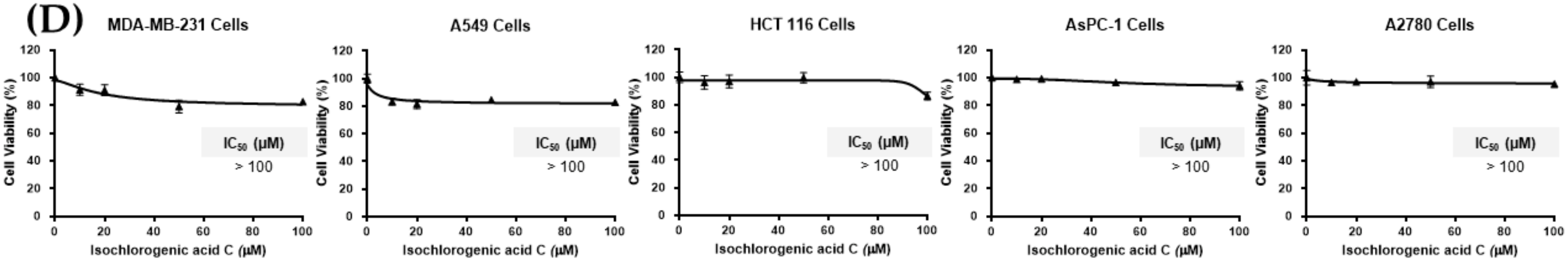
MDA-MB-231 Cells
A549 Cells
HCT 116 Cells
AsPC-1 Cells
A2780 Cells
Cell Viability (%)
Isochlorogenic acid C (μM)
IC50 (μM)
> 100

(E)

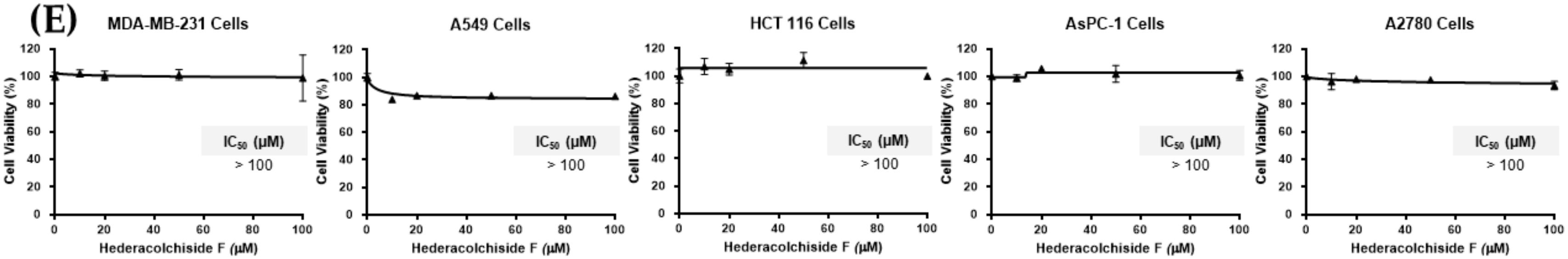
MDA-MB-231 Cells
A549 Cells
HCT 116 Cells
AsPC-1 Cells
A2780 Cells
Cell Viability (%)
Hederacolchiside F (μM)
IC50 (μM)
> 100

(F)

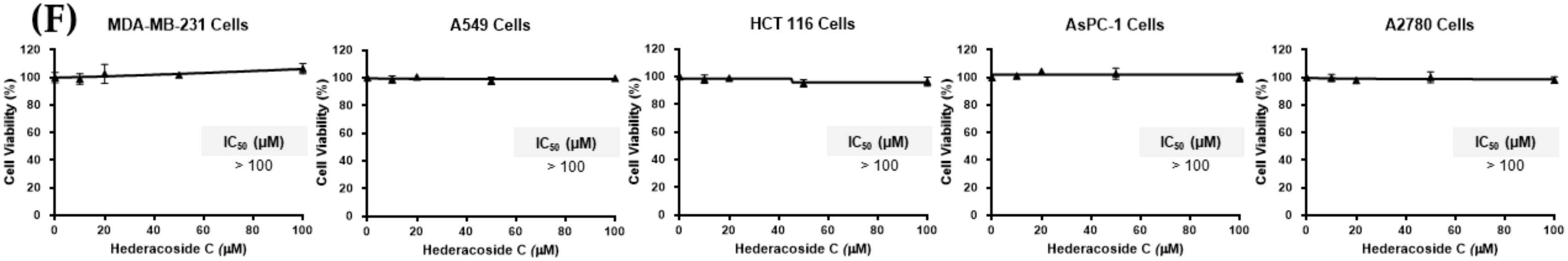
MDA-MB-231 Cells
A549 Cells
HCT 116 Cells
AsPC-1 Cells
A2780 Cells
Cell Viability (%)
Hederacoside C (μM)
IC50 (μM)
> 100

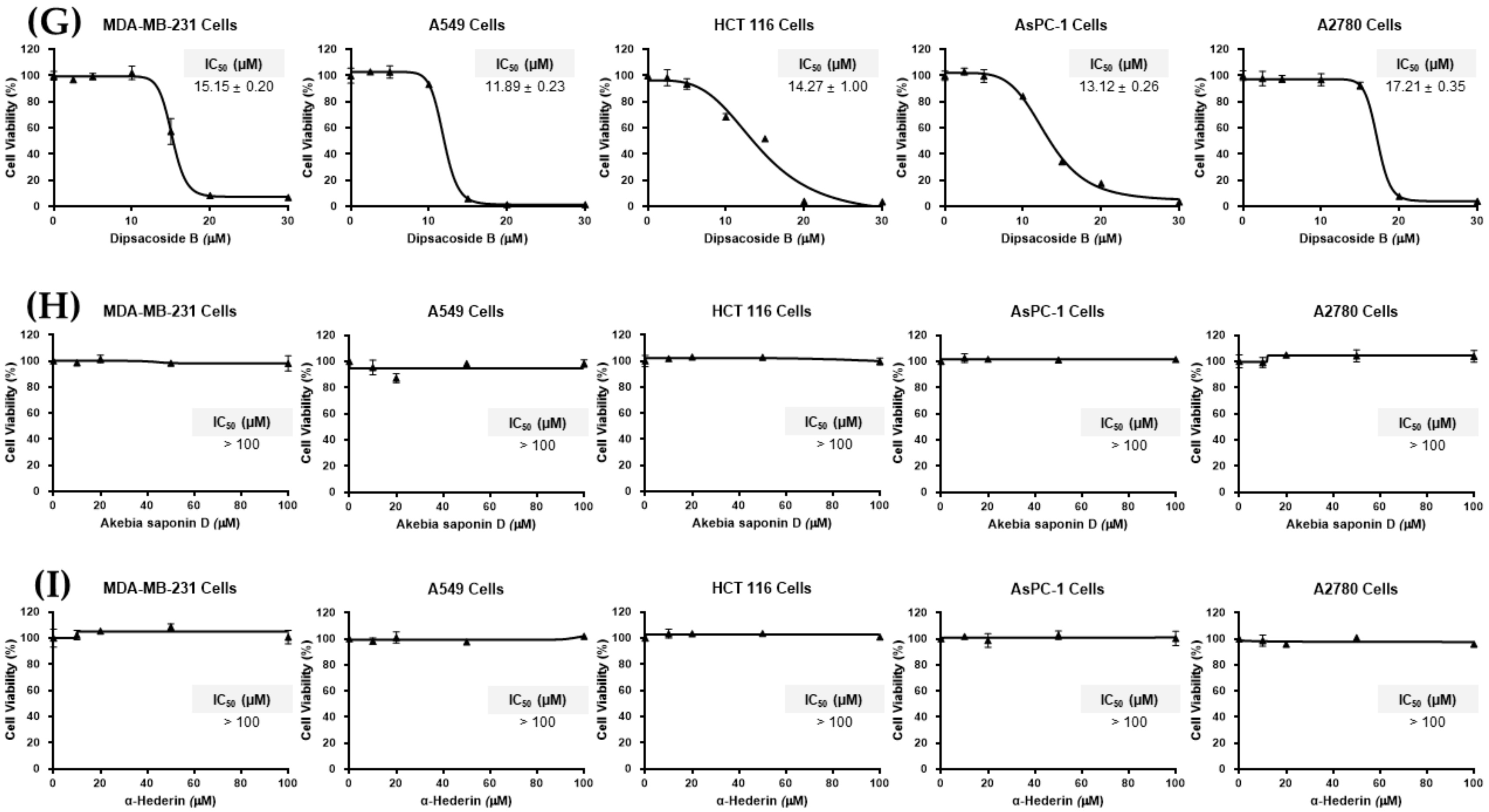
(G)
MDA-MB-231 Cells
A549 Cells
HCT 116 Cells
AsPC-1 Cells
A2780 Cells
IC50 (μM)
15.15 ± 0.20
11.89 ± 0.23
14.27 ± 1.00
13.12 ± 0.26
17.21 ± 0.35
Cell Viability (%)
Dipsacoside B (μM)
(H)
MDA-MB-231 Cells
A549 Cells
HCT 116 Cells
AsPC-1 Cells
A2780 Cells
IC50 (μM)
> 100
Akebia saponin D (μM)
(I)
MDA-MB-231 Cells
A549 Cells
HCT 116 Cells
AsPC-1 Cells
A2780 Cells
IC50 (μM)
> 100
α-Hederin (μM)

(J)

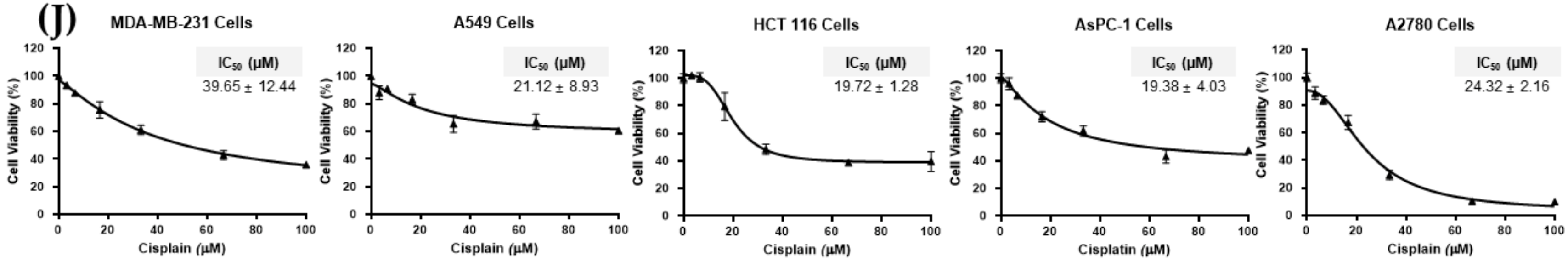

Figure S2. IC_{50} values of AQS extract (A), each marker compound (B–I), and positive control (J) in various human cell lines. AQS extract (A), chlorogenic acid (B), isochlorogenic acid A (C), isochlorogenic acid C (D), hederacolchiside F (E), hederacoside C (F), dipsacoside B (G), akebia saponin D (H), α-hederin (I), and positive control (cisplatin, J)

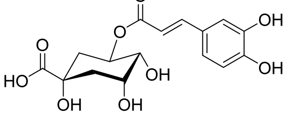

Chlorogenic acid

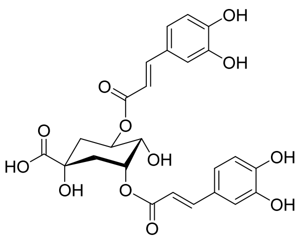

Isochlorogenic acid A

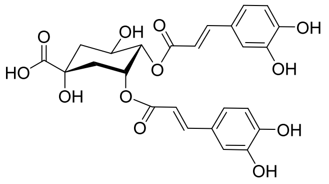

Isochlorogenic acid C

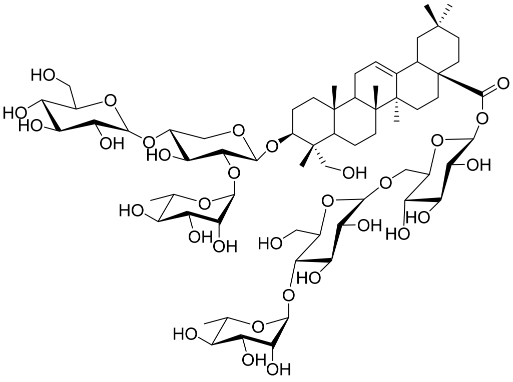

Hederacholichiside F

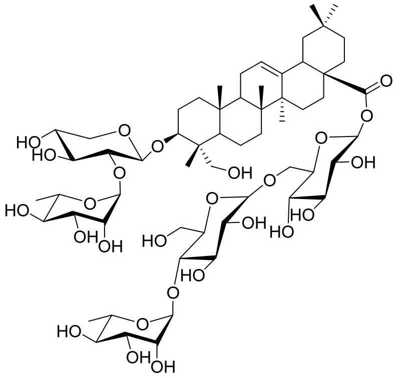

Hederacoside C

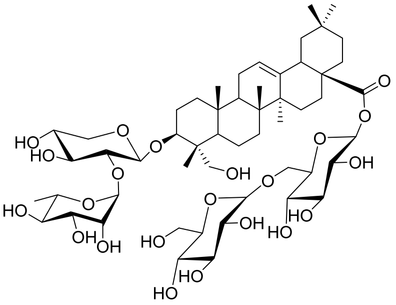

Dipsacoside B

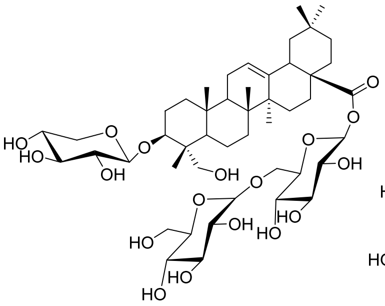

Akebia saponin D

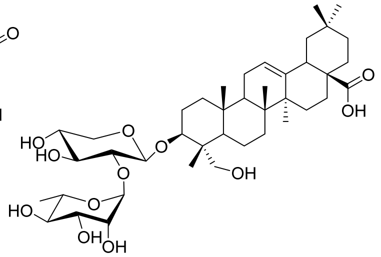

α-Hederin

Figure S3. Chemical structures of the target compounds selected for quality assessment of AQS sample.
